# Area Estimation of Deep-Sea Surfaces from Oblique Still Images

**DOI:** 10.1371/journal.pone.0133290

**Published:** 2015-07-15

**Authors:** Frederico Carvalho Dias, José Gomes-Pereira, Inês Tojeira, Miguel Souto, Andreia Afonso, António Calado, Pedro Madureira, Aldino Campos

**Affiliations:** 1 Task Group for the Extension of the Continental Shelf (EMEPC), Paço de Arcos, Portugal; 2 MARE—Marine and Environmental Sciences Centre, Departamento de Oceanografia e Pescas, Centre of IMAR, Universidade dos Açores, Horta, Portugal; Glasgow University, UNITED KINGDOM

## Abstract

Estimating the area of seabed surfaces from pictures or videos is an important problem in seafloor surveys. This task is complex to achieve with moving platforms such as submersibles, towed or remotely operated vehicles (ROV), where the recording camera is typically not static and provides an oblique view of the seafloor. A new method for obtaining seabed surface area estimates is presented here, using the classical set up of two laser devices fixed to the ROV frame projecting two parallel lines over the seabed. By combining lengths measured directly from the image containing the laser lines, the area of seabed surfaces is estimated, as well as the camera’s distance to the seabed, pan and tilt angles. The only parameters required are the distance between the parallel laser lines and the camera’s horizontal and vertical angles of view. The method was validated with a controlled *in situ* experiment using a deep-sea ROV, yielding an area estimate error of 1.5%. Further applications and generalizations of the method are discussed, with emphasis on deep-sea applications.

## Introduction

Estimating size, area and ground slope–besides distance–from photographic records is a long-lasting problem. During the last decades, the development and broad use of underwater vehicles or gear equipped with photographic and video cameras has turned this topic into a subject of major interest [1–3], applicable both to quantitative ecological studies [4–7], as well as to geological research and exploration efforts [8,9].

When an image is recorded with negligible optical distortion, which is generally the case in underwater visual surveys, proportions are preserved. This means that the lengths on the picture match with the corresponding angular sizes. The problem is then how to correlate angular size with “real” linear size and use the fact that angular size decreases with distance.

A straightforward approach has been to collect images using a camera that is perpendicular to the surface of interest, and employ a scaling object to obtain area measurements. This approach has the drawback of a restricted field of view and a limited applicability in underwater platforms that require a forward-facing camera for technical or navigational purposes [10,11]. A solution for estimating linear size in oblique images is to use a ruler next to the target of interest [3,4]. This, however, is applicable neither to non-stationary targets, e.g. in scenarios involving moving organisms, nor to recording of seabed features during navigation along a transect line. A major improvement to this technique, that has become the standard solution, is to use laser spots projected on the seabed or target at the time of image recording. In particular, the use of four [12] or more spots allows the estimation of surface area values [13]. Most of these methods rely on accurately knowing the height of the camera above the ground [14]. However, this parameter is not trivial to estimate for several platforms, e.g. towed cameras [15]. Typically, it is also required to keep the laser beams parallel to the optical axis of the camera [13], which is accomplished by coupling the laser devices to the camera.

Here, we present a new method for obtaining seabed area estimates in an oblique view image using two parallel laser lines that are fixed to the platform frame. Calculations and final simplifications are presented, including formulas to obtain camera distance from the seabed, and camera pan and tilt values. The results were validated with a controlled *in situ* experiment using a deep-sea ROV. Some applications of the method are discussed.

## Materials and Methods

**Fig 1:**
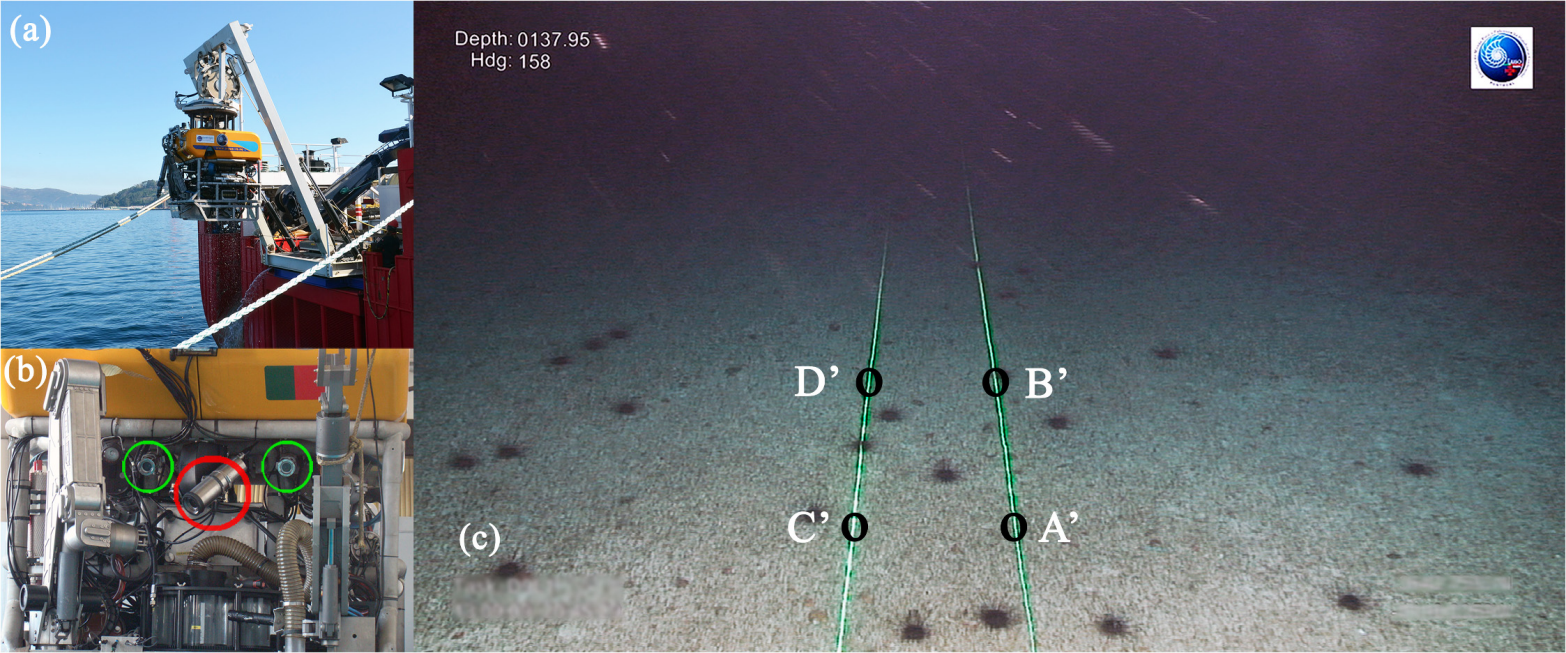
Surface and underwater view of the camera and laser optical system installed on the ROV. (a): Test launch of the ROV “Luso”. (b): Front view of the ROV, illustrating the HD camera (red circle) and the two laser devices (green circles). (c): Horizontal seabed still image containing the two (parallel) green laser lines; the trapezoid [A’B’D’C’] defines the surface area over the seafloor to be estimated.

Within the framework of the Portuguese Continental Shelf Extension Project, the ROV “Luso” has been in operation since 2008. It has surveyed and sampled the deep Northeast Atlantic seabed (Fig 1A). The ROV is a Deepwater Work Class, model Argus Bathysaurus XL rated for 6000 m depth (see reference [16] for technical details). It is owned and operated by EMEPC (Portuguese Task Group for the Extension of the Continental Shelf). Amongst other sensors, the ROV is equipped with a full HD video camera that allows recording footage of benthic environments from which still images are extracted. The camera is an Argus HD-SDI camera [17], comprising an Argus HD Camera Housing with a Sony FCB-H11 camera (10× optical zoom; focal distance 5.1–51mm). Fixed on the ROV frame, on both sides of the camera, there are two underwater line laser devices (Fig 1B), each generating a sharp green line (532 nm) over the seabed (Fig 1C). The two lasers are paralleled and calibrated at a set distance, generally *l* ∼ 60 cm apart. In this study, the value *l* = 67 cm was used for the distance between the laser lines. The laser lines do not move with respect to the ROV frame. Our mathematical solution is independent of any other specifics of the setup.

**Fig 2:**
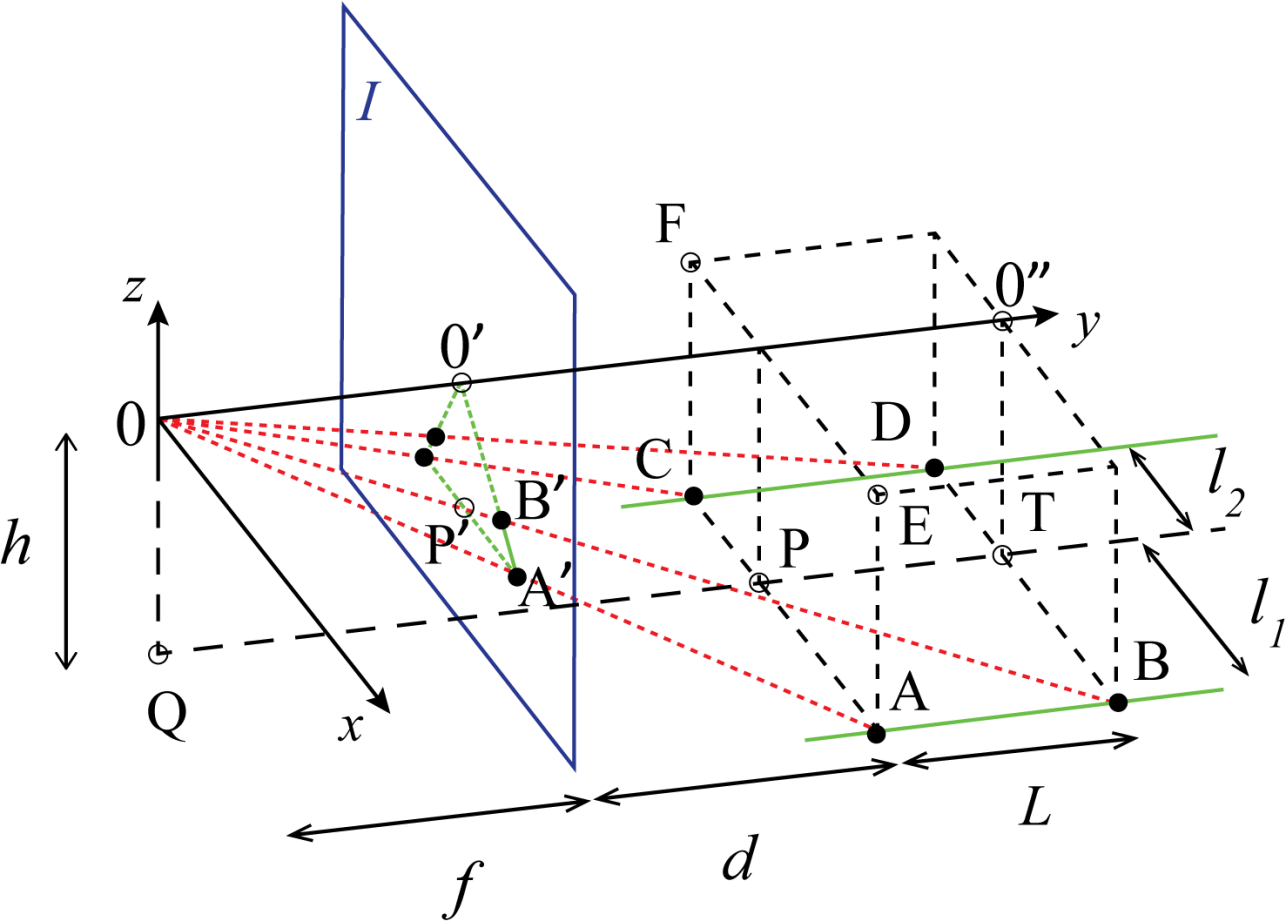
Three-dimensional geometry of the camera view with projected lasers for area calculation. Illustrated is the case of a horizontal camera positioned at the origin, with its optical axis (*y*-axis) being parallel to the green laser lines [AB] and [CD] on the horizontal seafloor. The laser lines are at a distance of *l* = *l*_1_ + *l*_2_ apart. The camera is located at a height *h* above the ground. Primed capital letters label the projections on the image *I* of the corresponding (unprimed) points on the seafloor. The red dotted lines represent light rays originating from points A, B, C, D and converging to the camera.

The objective is to calculate the seabed area *S* that corresponds to the trapezoid [A’B’D’C’] on the still picture (Fig 1C), given the distance *l* between the parallel laser lines. In other words, given the distance between the points A and C (or B and D) on the seabed (Fig 2), the distance *L* between A and B (or C and D) must be computed, by measuring the distances between the corresponding projected points A’ and B’ (or C’ and D’) on the still image, thus obtaining the area *S* = *L*×*l*.

In the Results and Discussion section we derive a mathematical solution to the problem that only involves elementary geometry and trigonometry. The equations are written in terms of distances in pixels that can be measured from the picture with an image analysis software such as ImageJ [18].

Our results were validated *in situ* during a test dive of the ROV “Luso”, from the Spanish R/V “Sarmiento de Gamboa” in the MOWER campaign (September 2014), for which no specific permits were required and that did not involve endangered or protected species. The dive was performed in the Western Mediterranean near the Bay of Cadiz, Spain at a depth of 643 m.

## Results and Discussion

The calculations presented in this section are based on the following assumptions: a) the seabed is horizontal; b) the laser devices are installed at both sides of the camera (Fig 1B); c) when the camera is aligned with the lasers, the projection [QT] on the seafloor of the optical axis [00”] is at distances *l*_1_ and *l*_2_ from the laser lines, with *l*_1_ + *l*_2_ = *l* (see Fig 2); d) the still image has negligible optical distortion and is not cropped; e) the camera’s horizontal and vertical angles of view, *α*_*H*_ and *α*_*V*_, respectively, are known; f) the center of the image coincides with the optical center.

Consider the special case of a camera with an optical axis that is horizontal and parallel to the laser lines (Fig 2). The camera is located at the origin, at a height *h* above the seabed. The plane of the image *I* is always perpendicular to the optical axis [00”] and lies between the camera and the virtual rectangle [ACFE], at an arbitrary distance *f* from the former. Thus, [ACFE] is at a distance *f* + *d* from the camera. The trapezoid [A’B’D’C’] projected on the image corresponds to the seabed rectangle [ABDC]. The vanishing point of the projected laser lines [A’B’] and [C’D’] coincides with the projection 0’ of the origin, i.e. the center of the image. In the camera’s reference frame (0*xyz* in Fig 2), the Cartesian coordinates of the seabed points A, B, C and D are given by

$$\begin{array}{cc} \{\begin{array}{l} x_{A}=l_{1}\\ y_{A}=f+d\\ z_{A}=-h \end{array} & \{\begin{array}{l} x_{B}=l_{1}\\ y_{B}=f+d+L\\ z_{A}=-h \end{array}\\ \{\begin{array}{l} x_{C}=-l_{2}\\ y_{C}=f+d\\ z_{C}=-h \end{array} & \{\begin{array}{l} x_{D}=-l_{2}\\ y_{D}=f+d+L\\ z_{D}=-h \end{array} \end{array}$$ (1)

The corresponding projections A’, B’, C’ and D’ are given by the intercept of the plane *I* with the line connecting the origin to the respective points on the seabed (the corresponding light rays, represented as red dotted lines in Fig 2). For example, the line segment [0A] is described by (*x*, *y*, *z*) = *t*(*x*_*A*_, *y*_*A*_, *z*_*A*_) with 0 ≤ *t* ≤ 1; for the point A’, *y*_*A*′_ = *f* applies, thus *t*_*A*′_ = *f* / *y*_*A*_, which allows to express *x*_*A*′_ and *z*_*A*′_ in terms of the coordinates of point A. Proceeding in the same way for the points B’, C’ and D’, it follows that

$$\begin{array}{cc} \{\begin{array}{l} x_{A\prime }=l_{1}f/(f+d)\\ z_{A\prime }=-hf/(f+d) \end{array} & \{\begin{array}{l} x_{B\prime }=l_{1}f/(f+d+L)\\ z_{B\prime }=-hf/(f+d+L) \end{array}\\ \{\begin{array}{l} x_{C\prime }=-l_{2}f/(f+d)\\ z_{C\prime }=-hf/(f+d) \end{array} & \{\begin{array}{l} x_{D\prime }=-l_{2}f/(f+d+L)\\ z_{D\prime }=-hf/(f+d+L) \end{array} \end{array}$$ (2)

where all *y*-coordinates are equal to *f*. Notice that the *x*- and *z*-coordinates of Eq (2) also give the points’ positions in the image relative to the image center 0’.

**Fig 3:**
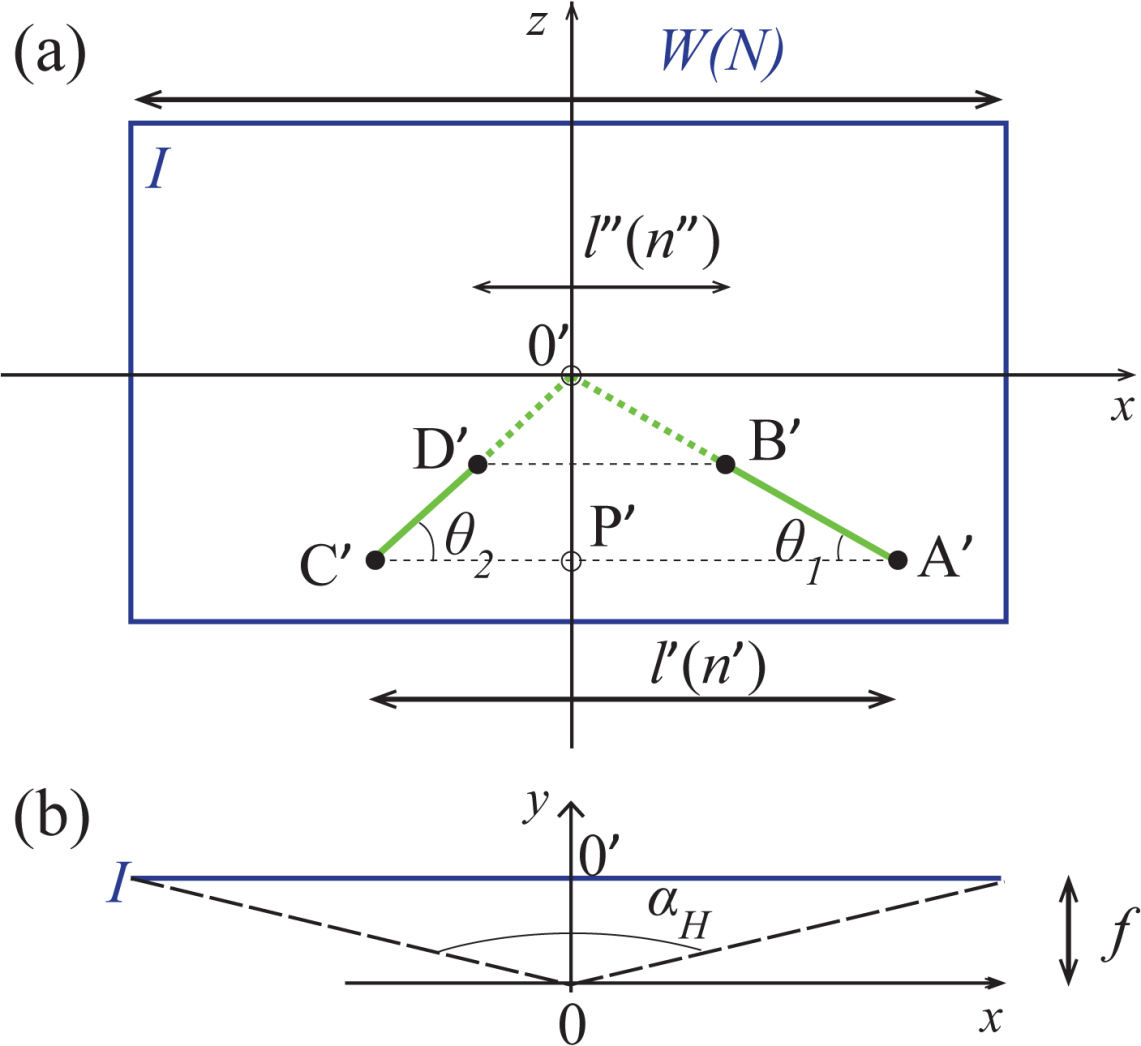
Projection of Fig 2 in two dimensions. (a): The image *I*. Notice that the *x*- and *z*-axis coincide with those of the camera, while the origin 0’ (image center) is the vanishing point for the laser lines. Variables inside parentheses correspond to the quantities expressed in pixels (e.g. the width *W* corresponds to *N* pixels). (b): Top view. The image forms at a distance *f* from the camera (at the origin 0), spanning a horizontal angle of view *α*_*H*_ over its width *W* (an analogous figure for the vertical angle of view *α*_*V*_ may be obtained by considering the 0*xz* plane).

The “perspective” angles *θ*_1_ and *θ*_2_ (see Fig 3A) are

$$\begin{array}{c} \text{cot}\;\theta_{1}=\frac{x_{A\prime }}{-z_{A\prime }}\\ \text{cot}\;\theta_{2}=\frac{-x_{C\prime }}{-z_{C\prime }} \end{array}$$ (3)

Note that there is no need to explicitly measure the "perspective" angles *θ*_1_ and *θ*_2_, although it is possible to do so with image analysis software [18]. In fact, Eq (3) shows that it suffices to determine length ratios from the still image in order to obtain the "perspective" angles (see Eq (23) for the general case in pixels).

By adding the two equalities in Eq (3) and using Eq (2), *h* becomes

$$h={\left(\text{cot}\;\theta_{1}+\text{cot}\;\theta_{2}\right)}^{-1}l$$ (4)

Let *l*′ = *x*_*A*′_ − *x*_*C*′_ be the length of segment [A’C’]. As an outcome of Eq (3), Fig 3A shows that

$$\text{cot}\;\theta_{1}+\text{cot}\;\theta_{2}=\frac{x_{A\prime }-x_{C\prime }}{-z_{A\prime }}=-\frac{l\prime }{z_{A\prime }}$$ (5)

applies to the "perspective" angles in (4). Therefore, by inserting this result into (4),

$$h=-\frac{z_{A\prime }}{l\prime }\times l$$ (6)

Eq (6) means that, given the distance *l* between the laser lines, the height of the camera above the seabed may be determined by the length ratio *z*_*A*′_ / *l*′ measured on the still image.

On the other hand, let the length of the segment [B’D’] be denoted by *l*″ = *x*_*B*′_ − *x*_*D*′_. By similarity of triangles [A0C] and [A’0C’], Fig 2 shows that

$$\frac{l}{\sqrt{y_{P}^{2}+z_{P}^{2}}}=\frac{l\prime }{\sqrt{y_{P\prime }^{2}+z_{P\prime }^{2}}}$$ (7)

Since *y*_*P*_ = *y*_*A*_, *z*_*P*_ = *z*_*A*_, *y*_*P*′_ = *y*_*A*′_, *z*_*P*′_ = *z*_*A*′_, by substituting into (7) the *y*- and *z*-coordinates given by Eqs (1) and (2), it follows that

$$\frac{l}{l\prime }=\frac{f+d}{f}$$ (8)

Analogously, for the triangles [B0D] and [B’0’D’] it results that

$$\frac{l}{l\prime \prime }=\frac{f+d+L}{f}$$ (9)

Combining (8) with (9) yields

$$L=\left(\frac{l\prime }{l\prime \prime }-1\right)\times f\times \frac{l}{l\prime }$$ (10)

Let *W* be the picture’s width, corresponding to the angular size *α*_*H*_ (see Fig 3B). Notice that *α*_*H*_ is the camera’s horizontal angle of view, which is assumed to be known, as is generally the case. Thereby

$$f=\frac{W}{2\;\text{tan}\left(\alpha_{H}\;/\;2\right)}$$ (11)

Eq (11) is always valid, even in the general case where the camera is rotated in relation to the ground, given that *f* is the distance to the image from the point of view of the camera.

By inserting (11) into (10), *L* becomes

$$L=\left(\frac{l\prime }{l\prime \prime }-1\right)\times \frac{W}{l\prime }\times \frac{l}{2\;\text{tan}(\alpha_{H}\;/\;2)}$$ (12)

Thus, assuming that the parameters *l* and *α*_*H*_ are known, the length *L* over the seafloor can be determined by length ratios measured on the still image.

Since the images are digital, *l*′, *l*″ and *W* are expressed as pixel counts. Let *N* be the horizontal size of the picture measured in pixels. Assuming that each pixel always corresponds to the same horizontal length *W* / *N*, let *n*′ be the number of pixels between A’ and C’ (Fig 3A). Analogously, let *n*″ be the number of pixels between B’ and D’ (Fig 3a). Thus, *l*′ = *n*′*W* / *N*, *l*″ = *n*″*W* / *N*, Eq (12) takes the form

$$L=\left(\frac{n\prime }{n\prime \prime }-1\right)\times \frac{N}{n\prime }\times \frac{l}{2\;\text{tan}(\alpha_{H}\;/\;2)}$$ (13)

and the area of the seabed surface corresponding to the trapezoid [A’B’D’C’] on the still image is

$$S=\left(\frac{n\prime }{n\prime \prime }-1\right)\times \frac{N}{n\prime }\times \frac{l^{2}}{2\;\text{tan}(\alpha_{H}\;/\;2)}$$ (14)

Note that the horizontal length per pixel *W* / *N* does not need to be determined, as it does not appear in Eqs (13) and (14).

### Seabed surface area estimates accounting for camera pan and tilt

ROV seafloor surveys typically involve the bulk collection of imagery using a camera that is vertically tilted by an angle *λ*, while simultaneously rotated by a pan angle *γ*. Panning or tilting the camera moves the vanishing point of the laser lines away from the center of the image. As the camera tilts towards the ground, the vanishing point moves upward relative to the image center. A positive (negative) pan corresponds to a rotation to the right (left). This is in fact the case of Fig 1C, that corresponds to a negative value of the pan angle *γ*, with the camera tilted towards the seabed. Presented below are only the main equations of interest for practical applications that result from generalizing the approach discussed in the previous section. The full details on the corresponding derivation can be found in S1 Text.

**Fig 4:**
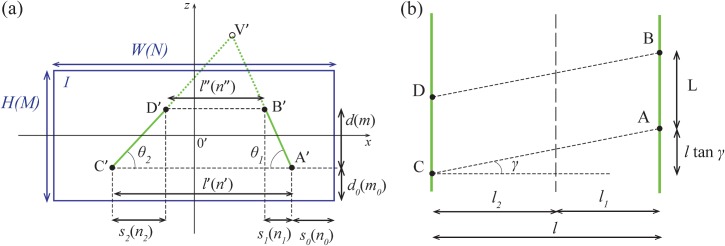
Geometry of a camera with a pan and tilt angle *γ* and *λ*, respectively. (a) The vanishing point V’ moves away from the center of the image. Note that V’ is shown outside the image frame only to remove clutter from the illustration. The variables inside parentheses represent the quantities in pixels. (b) The trapezoid [A’B’D’C’] on the image *I* corresponds to the seafloor parallelogram [ABDC]. The *y*-axis (not shown) is orthogonal to the lines [AC] and [BD]. The parallelogram [ABDC] has sides *l* / cos *λ* and *L*.

The coordinates of the vanishing point V’ in relation to the center of the image (see Fig 4A) are given by

$$x_{V\prime }=\frac{W}{2\;\text{tan}(\alpha_{H}\;/\;2)}\times \frac{\text{tan}\;\gamma }{\text{cos}\;\lambda }$$ (15)

and

$$z_{V\prime }=\frac{W}{2\;\text{tan}(\alpha_{H}\;/\;2)}\times \text{tan}\;\lambda$$ (16)

Eqs (15) and (16) show that the still images with the laser lines contain all the necessary information to determine the camera’s pan and tilt angles. In fact, from (16) one can estimate *λ* and then use (15) to get *γ* (see Eqs (20) and (21) below). This makes it possible to determine the length *L* over the seafloor for the general case (Fig 4),

$$L=\left(\frac{l\prime }{l\prime \prime }-1\right)\times \frac{W}{l\prime }\times \frac{l}{2\;\text{tan}(\alpha_{H}\;/\;2)\text{cos}\;\lambda \text{cos}\;\gamma }$$ (17)

Since *l*′ = *n*′*W* / *N* and *l*″ = *n*″*W* / *N* (see Fig 4A), Eq (16) becomes

$$L=\left(\frac{n\prime }{n\prime \prime }-1\right)\times \frac{N}{n\prime }\times \frac{l}{2\;\text{tan}(\alpha_{H}\;/\;2)\text{cos}\;\lambda \text{cos}\;\gamma }$$ (18)

where cos *λ* and cos *λ* are given below by Eqs (20) and (21), respectively.

For a non-zero value of the pan angle *γ* the trapezoid [A’B’D’C’] now defines a parallelogram of sides *l* / cos *γ* and *L* on the seafloor (see Fig 4B), as opposed to a rectangle for the case *γ* = 0 (see Fig 2). Nonetheless, the corresponding surface area is still given by *S* = *L*×*l*, which in pixel quantities (see Fig 4A) yields

$$S=\left(\frac{n\prime }{n\prime \prime }-1\right)\times \frac{N}{n\prime }\times \frac{l^{2}}{2\;\text{tan}(\alpha_{H}\;/\;2)\text{cos}\;\lambda \text{cos}\;\gamma }$$ (19)

where the effects of the camera’s tilt and pan are accounted by

$$\text{cos}\;\lambda ={\left\{1+{\left[\left(\frac{m_{0}}{M}-\frac{1}{2}+\frac{n\prime }{n_{1}+n_{2}}\times \frac{m}{M}\right)\times 2\;\text{tan}(\alpha_{V}\;/\;2)\right]}^{2}\right\}}^{-1/2}$$ (20)

and

$$\text{cos}\;\gamma ={\left\{1+{\left[\left(\frac{1}{2}-\frac{n_{0}}{N}-\frac{n_{1}}{n_{1}+n_{2}}\times \frac{n\prime }{N}\right)\times 2\;\text{tan}(\alpha_{H}\;/\;2)\text{cos}\;\lambda \right]}^{2}\right\}}^{-1/2}$$ (21)

Eqs (20) and (21) are expressed in terms of ratios of quantities directly obtainable from the still image (see Fig 4A). The camera’s vertical angle of view *α*_*V*_ appears in (20) because of the conversion of vertical linear dimensions into pixels.

### Camera distance to the seabed

The general version of Eq (4) gives the vertical distance to the seabed:

$$h=\frac{\text{cos}\;\lambda }{\text{cos}\;\gamma }{\left(\text{cot}\;\theta_{1}+\text{cot}\;\theta_{2}\right)}^{-1}\;l$$ (22)

with

$$\begin{array}{l} \text{cot}\;\theta_{1}=\frac{n_{1}}{N}\times \frac{M}{m}\times \frac{\text{tan}(\alpha_{H}\;/\;2)}{\text{tan}(\alpha_{V}\;/\;2)}\\ \text{cot}\;\theta_{2}=\frac{n_{2}}{N}\times \frac{M}{m}\times \frac{\text{tan}(\alpha_{H}\;/\;2)}{\text{tan}(\alpha_{V}\;/\;2)} \end{array}$$ (23)

Therefore, by using (20), (21) and (23) together with Eq (22), the height of the camera relative to the seafloor may be determined by pixel length ratios measured on the picture.

### Estimation of uncertainties

Consider Eq (17) for the length *L* over the seafloor. The value of *L* is a function of the measured lengths *l*′ and *l*″, the cosines of *γ* and *λ*, and the distance *l* between the laser lines. The uncertainties in these quantities generate an uncertainty Δ*L* in the value of *L*. The uncertainties in the camera's angles of view *α*_*H*_ and *α*_*V*_ are assumed to be negligible.

The maximum value of Δ*L* may be estimated by [19]

$$\Delta L=\left|\frac{\partial L}{\partial l\prime }\right|\Delta l\prime +\left|\frac{\partial L}{\partial l\prime \prime }\right|\Delta l\prime \prime +\left|\frac{\partial L}{\partial \text{cos}\;\gamma }\right|\Delta \text{cos}\;\gamma +\left|\frac{\partial L}{\partial \text{cos}\;\lambda }\right|\Delta \text{cos}\;\lambda +\left|\frac{\partial L}{\partial l}\right|\Delta l$$ (24)

where Δ*l*′, Δ*l*″, Δcos *γ*, Δcos *λ* and Δ*l* are the uncertainties in *l*′, *l*″, cos *γ*, cos *λ* and *l*, respectively. Typically, Δ*l* ∼ 0.5 cm is verified. Measuring lengths in pixels, e.g. *l*′ = *n*′*W* / *N*, implies Δ*l*′ = Δ*l*″ = *W* / *N*. Thus, using Eqs (17) and (18) together with Eq (24), the relative uncertainty in the value of *L* is

$$\frac{\Delta L}{L}=\left(\frac{n\prime \prime }{n\prime }+\frac{n\prime }{n\prime \prime }\right)\times \frac{1}{n\prime -n\prime \prime }+\frac{\Delta \text{cos}\;\gamma }{\text{cos}\;\gamma }+\frac{\Delta \text{cos}\;\lambda }{\text{cos}\;\lambda }+\frac{\Delta l}{l}$$ (25)

Since cos *γ* and cos *λ* are also determined by measurements taken from the still image, the corresponding relative uncertainties are obtained by using equations analogous to (24). From Eqs (20) and (21) it follows that

$$\begin{array}{l} \frac{\Delta \text{cos}\;\gamma }{\text{cos}\;\gamma }={\left[2\;\text{tan}\;\left(\alpha_{H}\;/\;2\right)\text{cos}\;\lambda \text{cos}\;\gamma \right]}^{2}\times \left|\frac{1}{2}-\frac{n_{0}}{N}-\frac{n_{1}}{n_{1}+n_{2}}\times \frac{n\prime }{N}\right|\\ \times \left\{\left[1+\left(1+2\frac{n_{1}}{n_{1}+n_{2}}\right)\times \frac{n\prime }{n_{1}+n_{2}}\right]\times \frac{1}{N}+\left|\frac{1}{2}-\frac{n_{0}}{N}-\frac{n_{1}}{n_{1}+n_{2}}\times \frac{n\prime }{N}\right|\times \frac{\Delta \text{cos}\;\lambda }{\text{cos}\;\lambda }\right\} \end{array}$$ (26)

and

$$\begin{array}{l} \frac{\Delta \text{cos}\;\lambda }{\text{cos}\;\lambda }=\left|\frac{m_{0}}{M}-\frac{1}{2}+\frac{n\prime }{n_{1}+n_{2}}\times \frac{m}{M}\right|\times {\left[2\;\text{tan}\;\left(\alpha_{V}\;/\;2\right)\text{cos}\;\lambda \right]}^{2}\\ \times \left[\left(1+\frac{n\prime }{n_{1}+n_{2}}\right)\times \frac{1}{M}+\left(1+2\frac{n\prime }{n_{1}+n_{2}}\right)\times \frac{1}{n_{1}+n_{2}}\right] \end{array}$$ (27)

Likewise, from (19) it follows that the relative uncertainty in *S* is

$$\frac{\Delta S}{S}=\left(\frac{n\prime \prime }{n\prime }+\frac{n\prime }{n\prime \prime }\right)\times \frac{1}{n\prime -n\prime \prime }+\frac{\Delta \text{cos}\;\gamma }{\text{cos}\;\gamma }+\frac{\Delta \text{cos}\;\lambda }{\text{cos}\;\lambda }+2\frac{\Delta l}{l}$$ (28)

and from (22) and (23) it results that the relative uncertainty in *h* is

$$\frac{\Delta h}{h}=\frac{1}{m}+\frac{2}{n_{1}+n_{2}}+\frac{\Delta \text{cos}\;\gamma }{\text{cos}\;\gamma }+\frac{\Delta \text{cos}\;\lambda }{\text{cos}\;\lambda }+\frac{\Delta l}{l}$$ (29)

where the relative uncertainties in cos *γ* and cos *λ* are given by Eqs (26) and (27), respectively.

### *In situ* validation

**Fig 5:**
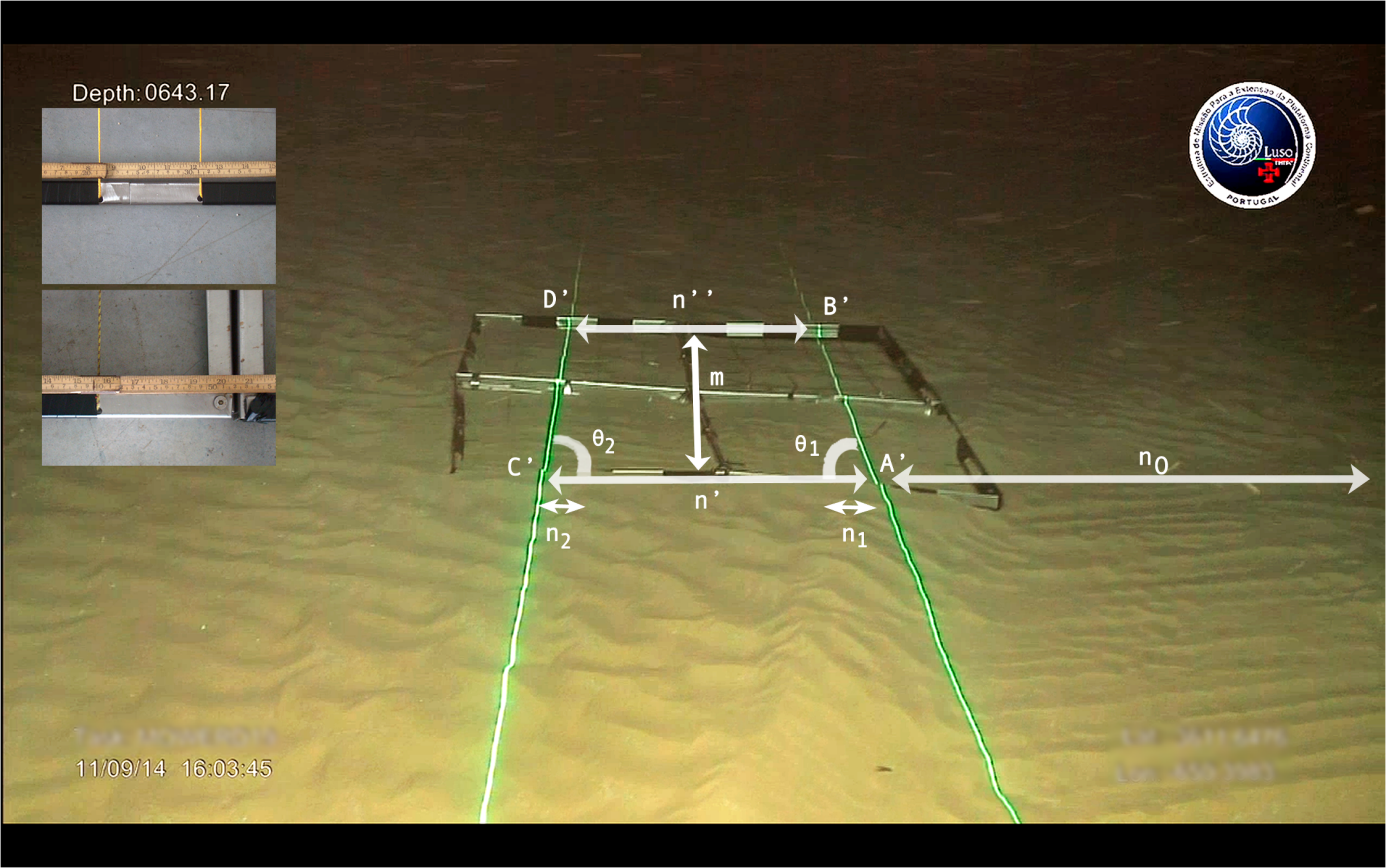
*In situ* validation using a known area. The trapezoid [A’B’D’C’] corresponds to an area of *S*_exp_ = 7236 ± 255 cm^2^ (*L*_exp_ = 108 ± 3 cm) over the seafloor. Inset: measurement of the size of the quadrat frames' inner grid and edge thickness.

*In situ* validation of our results was performed with the ROV "Luso", at a depth of 643 m (Fig 5). Although our mathematical solution does not depend on depth, in practice it is easier to properly resolve the laser lines in deep waters. At depths greater than ~100 m it is possible to avoid adverse shallow-water optical conditions such as bright ambient lighting and light scattering by suspended particulate matter.

Specifically, four identical quadrat frames were assembled, each with inner dimensions 50×50 cm and edge thickness 2.5 cm. Each square featured an inner grid of 10 cm. Thus, a 110×110 cm square object was deployed. Due to time and operational constraints, only one test dive was performed. All frames available from the existing footage are equivalent for the purposes of testing Eqs (19) and (22). Therefore, a single image was analyzed (Fig 5) and the area of the object located between the parallel laser lines was estimated. The still image was acquired with the ROV at rest on the seafloor. The height of the camera relative to the ground is *h*_exp_ = 113 ± 1 cm and the laser lines are *l* = 67 ± 0.5 cm apart. The horizontal and vertical angles of view for the still image are *α*_*H*_ = 50.43° and *α*_*V*_ = 29.67°, respectively.

Fig 5 shows that the test frame is slightly deformed due to the influence of the terrain conditions on the assembly geometry. This effect is accounted by considering that the linear distortion of the frame results in *L*_exp_ = 108 ± 3 cm. Thus, the trapezoid [A’B’D’C’] corresponds to an area of *S*_exp_ = *L*_exp_ × *l* = 7236 cm^2^. The uncertainty in *S*_exp_ is estimated from Δ*S*_exp_ / *S*_exp_ = Δ*L*_exp_ / *L*_exp_ + Δ*l* / *l*, which yields Δ*S*_exp_ = 255 cm^2^ (i.e. a percentage uncertainty of 3.5%).

The picture has a width of *N* = 1280 pixels and a height of *M* = 800 pixels. The following values (all in pixels) were measured from the still image using the software ImageJ [18]: *n*′ = 312, *n*″ = 232, *n*_0_ = 465, *n*_1_ = 52, *n*_2_ = 29, *m*_0_ = 367 and *m* = 143 (see Figs 4 and 5).

Given these measurements, Eq (18) yields *L* = 106.4 cm, affected by an uncertainty Δ*L* = 5.5 cm estimated from (25). As for the area of the seabed surface corresponding to the trapezoid [A’B’D’C’], from Eqs (19) and (28) it results that *S* = 71230 cm^2^ with an uncertainty Δ*S* = 425 cm^2^. These values are in excellent agreement with the respective experimental values. In fact, *L* differs ∼1.5% from *L*_exp_ and *S* differs ∼1.5% from *S*_exp_.

From Eq (22) it follows that the height of the camera relative to the seafloor is *h* = 100.7 cm, and (29) yields Δ*h* = 5.8 cm for the uncertainty. This is still an acceptable result, as *h* differs ∼11% from the true value *h*_exp_.

## Conclusions

The common usage of laser scaling devices in underwater image platforms is to have the lasers moving with the camera. This is generally the case for spot generating laser beams. Such a configuration is advantageous when estimating the dimensions of specific conspicuous objects or macro-organisms, as the camera and the laser spots are directed towards the target. However, it does not allow obtaining wide field area estimates.

This was achieved by [13] using five laser diodes with the Automated Benthic Image-Scaling System (ABISS). The method involves aligning four laser diodes in parallel to project the corners of a square of known dimensions, and placing a fifth unaligned laser in the same plane as the bottom pair of parallel lasers. The setup allows to calculate the camera to object distance. As camera orientation with respect to the substratum changes, the parallel laser spots projected onto the seabed appear as a trapezium. Finally, the *Benthic Imager* software (University of Plymouth, UK) calculates the actual seabed area contained within an image [5]. However, the installation of the five lasers around the camera lens can be complex, and a trigonometry solving software is required [5,13]. Lasers are often separated by short distances, and for wide angle views or as the recording platform increases the distance from the seafloor, the laser spots tend to disappear from sight, due to the relatively short distance over which visible light is absorbed. Finally, using no tilt, the laser beams will not be projected on the seafloor.

These constraints are overcome with the method that we present in this work. The two laser lines are simply projected over the seafloor and fixed to the ROV frame, thus not rotating with the camera. In most cases the two laser lines will be within sight, although dislocated due to the camera’s rotation, as described above. The use of laser lines instead of spots has the advantage of being easier to detect on still images, even in shallow water operations when scattered day light generally implies image filtering and processing to detect laser spots.

The seafloor area corresponding to a trapezoid defined by the laser lines in a still image is estimated directly from the picture by using Eq (19). Eq (22) yields the camera’s height above the ground, an important variable when estimating the accuracy of conspicuous target discrimination. The lengths in pixels can be measured using any standard image analysis software.

It is important to underline that our results depend on the validity of the assumptions used for their derivation, presented in the Results and Discussion section. The mathematical solution that we obtained breaks down if either the laser lines are not parallel, the camera produces non-negligible optical distortion or the still image is cropped. Two other parameters are needed, the camera’s horizontal and vertical angles of view, *α*_*H*_ and *α*_*V*_, respectively (or, in fact, the corresponding nonlinear zoom-dependent curves). They are generally available in the camera specifications or can be obtained from the manufacturer.

For the *in situ* experiment that was performed, the test area was estimated with an error of ∼1.5%. The excellent agreement between the calculated and the true values strongly supports the validity of our results. On the other hand, the height of the camera relative to the seafloor was estimated with an error of ∼11%. Although this is still an acceptable error for most situations, the magnitude of the discrepancy is almost an order of magnitude greater than for the case of the area estimation. This is due to the fact that the value calculated for *h* is especially sensible to the correct alignment of the laser lines, as it explicitly depends on the "perspective" angles *θ*_1_ and *θ*_2_ –see Eq (22). In practice, perfect parallelism between the laser lines will always be difficult to achieve. Therefore our solution will tend to produce a larger error for *h* than for *S*. This is not problematic, as the variable of interest is the area *S*. If necessary, the height *h* may be more accurately determined with help of one of several commercially available sensors, e.g. a standard altimeter.

As explained above, it was only possible to consider one independent image. Although the results of the experiment strongly support the robustness of our solution, it is of great importance to continue to perform similar tests. With a large enough data set it will be possible to determine the precision of the method with sound statistical tools. It is of particular interest to understand the sensitivity of the method to residual misalignments of the laser lines. In practice, these will be unavoidable. The results of the experiment suggest that the estimated area, Eq (19), is not significantly affected by such residual misalignments. A large data set encompassing different control objects will also allow to understand how the accuracy of the method depends on operational parameters such as distance to target, tilt and pan angles or ROV distance from the seabed.

The main equations only involve ratios between pixel lengths measured from the still image. Thus, it might be expected that there would be no significant change to the results if the picture were to be degraded to a lower resolution, as the rescaling factors would cancel out exactly in Eqs (18)–(23). However, coarse-graining effects might not be negligible, meaning that the values of the length ratios shall be affected. In other words, the rescaling factors will not cancel exactly. Nevertheless, the uncertainty in the calculated values may be estimated by using Eqs (25)–(29). These equations show that the uncertainties increase with decreasing image resolution, i.e. when *N* and *M* decrease. In fact, as *N* and *M* decrease, the length ratios in Eqs (25)–(29) approach unity, whereas the unbalanced terms will increase due to coarse-graining (e.g. 1 / (*n*′ − *n*″) in (25)).

The two parallel laser lines may be used as a natural scale in defining an equal seafloor area grid on the picture. By adapting the so called Canadian grid method [1], substituting the vanishing point V’ for the image’s optical center, it is straightforward to draw lines on the still image that correspond to virtual lines on the seafloor that are parallel to the visible laser lines.

The results presented in this work were derived under the assumption of a horizontal seabed. When such is not the case, the slope of the seafloor will be equivalent to an apparent tilt angle from the point of view of the camera. Therefore, if the actual tilt of the camera is known, the value of the apparent tilt angle will allow to correct for the inclination of the ground and to estimate the corresponding slope. The latter is a variable of great interest in benthic ecological studies and in geologic interpretation. For example, consider the camera facing a surface of inclination *α*, with the laser lines projected along the slope. If the camera is tilted downward by an angle *λ*_0_, Eq (20) gives an apparent tilt angle *λ* = *λ*_0_ + *α*. Thereby,

$$\alpha =\lambda -\lambda_{0}$$ (30)

Eq (30) shows that the seabed slope may be estimated by measuring the true tilt angle *λ*_0_. This can be achieved using a pan and tilt tracking sensor coupled to the camera. If relevant, a motion reference unit (mru) moving with the ROV may also be used to account for the attitude of the vehicle.

Note that Eq (19) still applies for the area *S* of a surface on a sloping seabed. In fact, the derivation of Eqs (15)–(29) remains unchanged when *λ* is interpreted as the angle between the camera's optical axis and the seabed surface. For a sloping seafloor *λ* = *λ*_0_ + *α* is the apparent tilt of the camera, whereas for a horizontal surface *λ* is the true vertical tilt angle.

The method presented in this work is independent of common sources of bias in transect methodologies for sampling the deep-sea, such as average field of view or transect distances. Therefore, it might have been useful in previous deep-sea projects that involved estimating seafloor densities, e.g. of benthic organisms [20–22], marine litter [23,24] or geologic structures [25]. It will be of use in future or ongoing projects (e.g. MIDAS [26] or JPI Oceans [27]), providing more accurate measurements from image data of seafloor surveys. Integration of this method into automated laser recognition routines could greatly enhance the technological capacity of seafloor assessments.

## Supporting Information

S1 Text Appendix. (DOCX)
