# Supplementary material for: Area Estimation of Deep-Sea Surfaces from Oblique Still Images
Source: PLoS One. 2015 Jul 15;10(7):e0133290. doi: 10.1371/journal.pone.0133290 (PMC4503614; doi:10.1371/journal.pone.0133290)
Supplement: S1 Text — (DOCX) [file pone.0133290.s001.docx]

# S1 Text. Appendix.

A rotation by an angle $\beta$ about an axis defined by the unit vector $\mathbf{k}=(k_{x},k_{y},k_{z})$ is geometrically described by the rotation matrix [1]

$$R_{\mathbf{k}}(\beta)=\left(\begin{matrix} \cos\beta+(1-\cos^{2}\beta)k_{x}^{2} & (1-\cos\beta)k_{x}k_{y}-k_{z}\sin\beta& (1-\cos\beta)k_{x}k_{z}+k_{y}\sin\beta\\ (1-\cos\beta)k_{x}k_{y}+k_{z}\sin\beta& \cos\beta+(1-\cos^{2}\beta)k_{y}^{2} & (1-\cos\beta)k_{y}k_{z}-k_{x}\sin\beta\\ (1-\cos\beta)k_{x}k_{z}-k_{y}\sin\beta& (1-\cos\beta)k_{y}k_{z}+k_{x}\sin\beta& \cos\beta+(1-\cos^{2}\beta)k_{z}^{2} \end{matrix}\right)$$

Let the $0xyz$ reference frame rotate with the camera. The optical axis defines the *y*-axis, with the camera at the origin. The pan axis is vertical and fixed. The *x*-axis coincides with the tilt axis, which is horizontal, pans with the camera and is always perpendicular to the camera's optical axis. The camera is horizontal at the starting position, as depicted in Fig. 2 in the main text.

Consider the case where the camera is vertically tilted by an angle $-\lambda$ (the minus sign means a downward tilt) and simultaneously rotated by a pan angle $\gamma$. From the point of view of the camera its orientation is described by a rotation $R_{\mathbf{u}}(\lambda)$ followed by a rotation $R_{R_{\mathbf{u}}(\lambda)\mathbf{v}}(-\gamma)$. The unit vector $\mathbf{u}=(1,0,0)$ defines the tilt axis, $\mathbf{v}=(0,0,1)$ is the *z*-axis unit vector and the unit vector $R_{\mathbf{u}}(\lambda)\mathbf{v}$ defines the vertical direction, i.e. the pan axis, after the tilt rotation. For example, point A, on the seafloor, has initial coordinates $(l_{1},y_{A0},-h)$; after the camera is rotated its coordinates become

$$\left(\begin{matrix} x_{A} \\ y_{A} \\ z_{A} \end{matrix}\right)=R_{R_{\mathbf{u}}(\lambda)\mathbf{v}}(-\gamma)R_{\mathbf{u}}(\lambda)\left(\begin{matrix} l_{1} \\ y_{A0} \\ -h \end{matrix}\right)=\left(\begin{matrix} \cos\gamma& \sin\gamma& 0 \\ -\sin\gamma\cos\lambda& \cos\gamma\cos\lambda& -\sin\lambda\\ -\sin\gamma\sin\lambda& \cos\gamma\sin\lambda& \cos\lambda\end{matrix}\right)\times\left(\begin{matrix} l_{1} \\ y_{A0} \\ -h \end{matrix}\right)$$

Proceeding analogously for points B, C and D, after the complete rotation the coordinates of the four seafloor points become

$$\begin{matrix} \left\{\begin{matrix} x_{A}=l_{1}\cos\gamma+y_{A0}\sin\gamma\\ y_{A}=-l_{1}\cos\lambda\sin\gamma+y_{A0}\cos\lambda\cos\gamma+h\sin\lambda\\ z_{A}=-l_{1}\sin\lambda\sin\gamma+y_{A0}\sin\lambda\cos\gamma-h\cos\lambda\end{matrix}\right. \\ \left\{\begin{matrix} x_{B}=l_{1}\cos\gamma+(y_{A0}+L_{1})\sin\gamma\\ y_{B}=-l_{1}\cos\lambda\sin\gamma+(y_{A0}+L_{1})\cos\lambda\cos\gamma+h\sin\lambda\\ z_{B}=-l_{1}\sin\lambda\sin\gamma+(y_{A0}+L_{1})\sin\lambda\cos\gamma-h\cos\lambda\end{matrix}\right. \\ \left\{\begin{matrix} x_{C}=-l_{2}\cos\gamma+y_{C0}\sin\gamma\\ y_{C}=l_{2}\cos\lambda\sin\gamma+y_{C0}\cos\lambda\cos\gamma+h\sin\lambda\\ z_{C}=l_{2}\sin\lambda\sin\gamma+y_{C0}\sin\lambda\cos\gamma-h\cos\lambda\end{matrix}\right. \\ \left\{\begin{matrix} x_{D}=-l_{2}\cos\gamma+(y_{C0}+L_{2})\sin\gamma\\ y_{D}=l_{2}\cos\lambda\sin\gamma+(y_{C0}+L_{2})\cos\lambda\cos\gamma+h\sin\lambda\\ z_{D}=l_{2}\sin\lambda\sin\gamma+(y_{C0}+L_{2})\sin\lambda\cos\gamma-h\cos\lambda\end{matrix}\right. \end{matrix}$$

Relative to equation (1) in the main text, equation accounts for the more general case where $y_{A0}$ may not be equal to $y_{C0}$ – both coordinates are equal to $f+d$ in (1). The distances $\overline{AB}=L_{1}$ and $\overline{CD}=L_{2}$ are also not necessarily the same. Since the image plane *I* remains at $y=f$, the coordinates for the projected points on the picture are obtained in a similar fashion as for equation (2) in the main text:

$$\begin{matrix} \left\{\begin{matrix} x_{A'}=f\frac{l_{1}\cos\gamma+y_{A0}\sin\gamma}{-l_{1}\cos\lambda\sin\gamma+y_{A0}\cos\lambda\cos\gamma+h\sin\lambda} \\ z_{A'}=f\frac{-l_{1}\sin\lambda\sin\gamma+y_{A0}\sin\lambda\cos\gamma-h\cos\lambda}{-l_{1}\cos\lambda\sin\gamma+y_{A0}\cos\lambda\cos\gamma+h\sin\lambda} \end{matrix}\right. \\ \left\{\begin{matrix} x_{B'}=f\frac{l_{1}\cos\gamma+(y_{A0}+L_{1})\sin\gamma}{-l_{1}\cos\lambda\sin\gamma+(y_{A0}+L_{1})\cos\lambda\cos\gamma+h\sin\lambda} \\ z_{B'}=f\frac{-l_{1}\sin\lambda\sin\gamma+(y_{A0}+L_{1})\sin\lambda\cos\gamma-h\cos\lambda}{-l_{1}\cos\lambda\sin\gamma+(y_{A0}+L_{1})\cos\lambda\cos\gamma+h\sin\lambda} \end{matrix}\right. \\ \left\{\begin{matrix} x_{C'}=f\frac{-l_{2}\cos\gamma+y_{C0}\sin\gamma}{l_{2}\cos\lambda\sin\gamma+y_{C0}\cos\lambda\cos\gamma+h\sin\lambda} \\ z_{C'}=f\frac{l_{2}\sin\lambda\sin\gamma+y_{C0}\sin\lambda\cos\gamma-h\cos\lambda}{l_{2}\cos\lambda\sin\gamma+y_{C0}\cos\lambda\cos\gamma+h\sin\lambda} \end{matrix}\right. \\ \left\{\begin{matrix} x_{D'}=f\frac{-l_{2}\cos\gamma+(y_{C0}+L_{2})\sin\gamma}{l_{2}\cos\lambda\sin\gamma+(y_{C0}+L_{2})\cos\lambda\cos\gamma+h\sin\lambda} \\ z_{D'}=f\frac{l_{2}\sin\lambda\sin\gamma+(y_{C0}+L_{2})\sin\lambda\cos\gamma-h\cos\lambda}{l_{2}\cos\lambda\sin\gamma+(y_{C0}+L_{2})\cos\lambda\cos\gamma+h\sin\lambda} \end{matrix}\right. \end{matrix}$$

The camera’s rotation moves the vanishing point of the projected laser lines relative to the image center to a new position $(x_{V'},z_{V'})$, as depicted in Fig. 4a in the main text. The vanishing point V’ is by definition the intersection point of the straight lines [A’B’] and [C’D’], described respectively by

$$\begin{matrix} z=-(x-x_{A'})/\cot\theta_{1}+z_{A'} \\ z=(x-x_{C'})/\cot\theta_{2}+z_{C'} \end{matrix}$$

where the “perspective” angles $\theta_{1}$ and $\theta_{2}$ (see Fig. 4a in the main text) are obtained by generalizing equation (3) in the main text to the present case,

$$\begin{matrix} \cot\theta_{1}=-\frac{x_{A'}-x_{B'}}{z_{A'}-z_{B'}}=\frac{l_{1}\cos\lambda-h\sin\lambda\sin\gamma}{h\cos\gamma} \\ \cot\theta_{2}=-\frac{x_{D'}-x_{C'}}{z_{D'}-z_{C'}}=\frac{l_{2}\cos\lambda+h\sin\lambda\sin\gamma}{h\cos\gamma} \end{matrix}$$

From it follows that $-(x_{V'}-x_{A'})/\cot\theta_{1}+z_{A'}=(x_{V'}-x_{C'})/\cot\theta_{2}+z_{C'}$ applies at V’. Solving for $x_{V'}$ and using together with , it results that

$$x_{V'}=f\frac{\tan\gamma}{\cos\lambda}$$

From equation (11) in the main text, inserting $f=W/2\tan\left(\alpha_{H}/2\right)$ into the last equation yields equation (15) in the main text:

$$x_{V'}=\frac{W}{2\tan(\alpha_{H}/2)}\times\frac{\tan\gamma}{\cos\lambda}$$

The *z*-coordinate of the vanishing point is obtained by inserting into either one of the equations in :

$$z_{V'}=\frac{W}{2\tan(\alpha_{H}/2)}\times\tan\lambda$$

Next, by adding the two equations in and solving for $h$, the general expression for the camera's height relative the ground, equation (22) in the main text, is obtained:

$$h=\frac{\cos\lambda}{\cos\gamma}\left(\cot\theta_{1}+\cot\theta_{2}\right)^{-1}l$$

If $z_{A'}=z_{C'}$ applies (as in Fig. 1c in the main text), it follows from that

$$y_{A0}-y_{C0}=l\tan\gamma$$

Analogously, if $z_{B'}=z_{D'}$ then

$$L_{1}=L_{2}$$

Thus, let $L=L_{1}=L_{2}$. From equations and follows that the trapezoid [A’B’D’C’] in Fig. 1c corresponds to a parallelogram [ABDC] on the seabed with sides $l/\cos\gamma$ and $L$ (see Fig. 4b in the main text) and inner angles $90\mathrm{º}\pm\gamma$. Accordingly, the area of [ABDC] is $S=L\times l$.

As for the special case of equation (8) in the main text, by similarity of triangles [A0C] and [A’0’C’] it follows that

$$\frac{l/\cos\gamma}{l'}=\frac{y_{A}}{f}$$

Note that $f+d$ in equation (8) corresponds to $y_{A}$ in and $l$ is exchanged for $l/\cos\gamma$.

From $y_{B}=y_{A}+L\cos\lambda\cos\gamma$ applies, thus, by similarity of triangles [B0D] and [B’0’D’], the general case of equation (9) is

$$\frac{l/\cos\gamma}{l''}=\frac{y_{A}+L\cos\lambda\cos\gamma}{f}$$

Combining and with (11) yields equation (17) in the main text:

$$L=\left(\frac{l'}{l''}-1\right)\times\frac{W}{l'}\times\frac{l}{2\tan(\alpha_{H}/2)\cos\lambda\cos\gamma}$$

In many cases the vanishing point V’ lies outside the picture, as in Fig. 1c in the main text. In such a situation, the coordinates $x_{V'}$ and $z_{V'}$ must be calculated, so that the cosines of the tilt and pan angles may be used in equation (19) in the main text. Therefore, consider the case depicted in Fig. 4a in the main text. At the vanishing point V’ equation becomes

$$\begin{matrix} x_{A'}-x_{V'}=(z_{V'}-z_{A'})\cot\theta_{1} \\ x_{V'}-x_{C'}=(z_{V'}-z_{C'})\cot\theta_{2} \end{matrix}$$

Adding the two equalities in and solving for $z_{V'}$ yields

$$z_{V'}=z_{A'}+\frac{l'}{\cot\theta_{1}+\cot\theta_{2}}$$

Since (see Fig. 4a in the main text)

$$\begin{matrix} \cot\theta_{1}=s_{1}/d \\ \cot\theta_{2}=s_{2}/d \end{matrix}$$

it follows that is equivalent to

$$z_{V'}=z_{A'}+\frac{l'}{s_{1}+s_{2}}d$$

Let $H$ be the picture's height. Analogously to equation (11) in the main text, $H$ obeys

$$f=\frac{H}{2\tan(\alpha_{V}/2)}$$

Thereby,

$$\frac{W}{2\tan(\alpha_{H}/2)}=\frac{H}{2\tan(\alpha_{V}/2)}$$

Inserting this result into equation and solving for $\cos\lambda$ yields

$$\cos\lambda=\left[1+\left(\frac{z_{V'}}{H/2\tan(\alpha_{V}/2)}\right)^{2}\right]^{-1/2}$$

Let $M$ be the vertical size of the picture in pixels, so that $H/M$ is the height per pixel. Hence, $d=mH/M$ and $z_{A'}=-\left(M/2-m_{0}\right)H/M$ (see Fig. 4a in the main text). Since $l'/\left(s_{1}+s_{2}\right)=n'/\left(n_{1}+n_{2}\right)$, equation becomes

$$\frac{z_{V'}}{H}=\frac{m_{0}}{M}-\frac{1}{2}+\frac{n'}{n_{1}+n_{2}}\times\frac{m}{M}$$

Inserting this equality into , the cosine of the tilt angle is expressed in terms of pixel quantities directly measurable from the picture,

$$\cos\lambda=\left\{1+\left[\left(\frac{m_{0}}{M}-\frac{1}{2}+\frac{n'}{n_{1}+n_{2}}\times\frac{m}{M}\right)\times2\tan(\alpha_{V}/2)\right]^{2}\right\}^{-1/2}$$

which is equation (20) in the main text. Similarly, $\cos\gamma$ may be obtained from equation :

$$\cos\gamma=\left[1+\left(\frac{x_{V'}}{W}\times2\tan(\alpha_{H}/2)\cos\lambda\right)^{2}\right]^{-1/2}$$

Subtracting the two equalities in equation and solving for $x_{V'}$, it follows that

$$x_{V'}=x_{A'}-\frac{\cot\theta_{1}}{\cot\theta_{1}+\cot\theta_{2}}$$

which, by the use of , is equivalent to

$$x_{V'}=x_{A'}-\frac{s_{1}}{s_{1}+s_{2}}l'$$

Using quantities in pixels (see Fig. 4a in the main text), this last equation becomes

$$\frac{x_{V'}}{W}=\frac{1}{2}-\frac{n_{0}}{N}-\frac{n_{1}}{n_{1}+n_{2}}\times\frac{n'}{N}$$

Substituting $x_{V'}/W$ into , equation (21) in the main text is obtained:

$$\cos\gamma=\left\{1+\left[\left(\frac{1}{2}-\frac{n_{0}}{N}-\frac{n_{1}}{n_{1}+n_{2}}\times\frac{n'}{N}\right)\times2\tan\left(\alpha_{H}/2\right)\cos\lambda\right]^{2}\right\}^{-1/2}$$

Note that the procedure for obtaining $\cos\lambda$ and $\cos\gamma$ (equations and , respectively) remains valid when V’ is inside the image.

# References

1. Elliott JP, Dawber PG. Symmetry in Physics. Vol.1: Principles and Simple Applications. MacMillan; 1990.
